# Clinical correlations with *Porphyromonas gingivalis *antibody responses in patients with early rheumatoid arthritis

**DOI:** 10.1186/ar4289

**Published:** 2013-09-09

**Authors:** Sheila L Arvikar, Deborah S Collier, Mark C Fisher, Sebastian Unizony, George L Cohen, Gail McHugh, Toshihisa Kawai, Klemen Strle, Allen C Steere

**Affiliations:** 1 Division of Rheumatology, Allergy and Immunology, Center for Immunology and Inflammatory Diseases, Massachusetts General Hospital, Harvard Medical School, 149 13th Street, Charlestown, MA 02129, USA; 2 The Forsyth Institute, 245 First Street, Cambridge, MA 02142, USA; 3 Center for Immunology and Inflammatory Diseases, CNY149/8301, 149 13th Street, Charlestown, MA 02129

## Abstract

**Introduction:**

Prior studies have demonstrated an increased frequency of antibodies to *Porphyromonas gingivalis *(*Pg*), a leading agent of periodontal disease, in rheumatoid arthritis (RA) patients. However, these patients generally had long-standing disease, and clinical associations with these antibodies were inconsistent. Our goal was to examine *Pg *antibody responses and their clinical associations in patients with early RA prior to and after disease-modifying antirheumatic drug (DMARD) therapy.

**Methods:**

Serum samples from 50 DMARD-naïve RA patients were tested using an enzyme-linked immunosorbent assay with whole-*Pg *sonicate. For comparison, serum samples were tested from patients with late RA, patients with other connective tissue diseases (CTDs), age-similar healthy hospital personnel and blood bank donors. *Pg *antibody responses in early RA patients were correlated with standard RA biomarkers, measures of disease activity and function.

**Results:**

At the time of enrollment, 17 (34%) of the 50 patients with early RA had positive immunoglobulin G (IgG) antibody responses to *Pg*, as did 13 (30%) of the 43 patients with late RA. RA patients had significantly higher *Pg *antibody responses than healthy hospital personnel and blood bank donors (*P *< 0.0001). Additionally, RA patients tended to have higher *Pg *antibody reactivity than patients with other CTDs (*P *= 0.1), and CTD patients tended to have higher *Pg *responses than healthy participants (*P *= 0.07). Compared with *Pg *antibody-negative patients, early RA patients with positive *Pg *responses more often had anti-cyclic citrullinated peptide (anti-CCP) antibody reactivity, their anti-CCP levels were significantly higher (*P *= 0.03) and the levels of anti-*Pg *antibodies correlated directly with anti-CCP levels (*P *< 0.01). Furthermore, at the time of study entry, the *Pg*-positive antibody group had greater rheumatoid factor values (*P *= 0.04) and higher inflammatory markers (erythrocyte sedimentation rate, or ESR) (*P *= 0.05), and they tended to have higher disease activity scores (Disease Activity Score based on 28-joint count (DAS28)-ESR and Clinical Disease Activity Index) and more functional impairment (Health Assessment Questionnaire). In *Pg*-positive patients, greater disease activity was still apparent after 12 months of DMARD therapy.

**Conclusions:**

A subset of early RA patients had positive *Pg *antibody responses. The responses correlated with anti-CCP antibody reactivity and to a lesser degree with ESR values. There was a trend toward greater disease activity in *Pg*-positive patients, and this trend remained after 12 months of DMARD therapy. These findings are consistent with a role for *Pg *in disease pathogenesis in a subset of RA patients.

## Introduction

The etiology of rheumatoid arthritis (RA) is unknown, but both environmental and genetic factors are likely to play roles in its pathogenesis. Periodontal disease (PD), an inflammatory disease of tooth-supporting structures, may be an environmental trigger for RA. Compared with healthy controls, PD is more frequent in RA patients, both in those with new-onset and in those with long-standing disease, even when potential confounding factors such as smoking are taken into account [1-5].

Furthermore, there is increasing evidence of a role for PD pathogens, particularly *Porphyromonas gingivalis *(*Pg*), in RA pathogenesis. *Pg *is the only prokaryote known to possess a peptidylarginine deiminase (PAD), an enzyme that catalyzes the posttranslational modification of arginine residues to citrulline. Although citrullination may occur more generally in sites of inflammation, antibodies to citrullinated proteins (anti-cyclic citrullinated peptide (anti-CCP) antibodies) are specific for RA and are now valuable diagnostic markers for the disease [6]. CCP antibodies are associated with a more aggressive course [7] and may be detected prior to the onset of clinical disease [8], suggesting a role in RA pathogenesis. *Pg*, through its PAD enzyme, may citrullinate host or bacterial proteins [9], altering their antigenicity and triggering autoimmunity and RA in predisposed individuals [9,10]. Further support for this hypothesis comes from animal models. *Pg *enolase has been found to cause arthritis in DR4-IE-transgenic mice [11], and *Pg *infection has been shown to exacerbate collagen antibody-induced arthritis [12].

Prior studies have demonstrated increased frequencies of antibody responses to *Pg *in RA patients compared with healthy controls [5,13-16]. However, in these studies [5,13,15,16], patients generally had long-standing disease and were presumably receiving disease-modifying antirheumatic drugs (DMARDs), factors which may affect infection with PD pathogens and serum antibody responses. Moreover, clinical correlations with *Pg *responses have been inconsistent. Some investigators have noted an association of *Pg *antibodies with anti-CCP antibody levels, but not with RF values [14,15], whereas others found a correlation of *Pg *immunoglobulin G (IgG) antibodies with RF levels, but not with CCP antibody values [5,16]. Additionally, some researchers found an association of *Pg *antibodies and elevated C-reactive protein (CRP) levels [13,14], but others noted no correlation between these antibodies and Disease Activity Score based on 28-joint count (DAS28)-CRP values [16].

Patients with early RA are an important group to study because they may benefit the most from treatment interventions for *Pg *and PD. In the only previous study in which *Pg *antibodies were examined in early RA patients, Scher *et al*. [4] found no significant difference in antibodies to a specific *Pg *antigen (*Pg*-specific chaperone protein HtpG) in patients with new-onset or chronic RA compared with control participants. However, antibody responses to whole-*Pg *antigen preparations have not yet been reported in early RA patients. Furthermore, *Pg *antibody responses in RA patients have not been compared to those of patients with other connective tissue diseases (CTDs), such as lupus, which are associated with broad immune stimulation.

In this study, we determined *Pg *antibody responses in patients with early RA and in comparison groups and correlated these results with standard RA biomarkers, disease activity scores and measures of function. We report here that both early and late RA patients had significantly more frequent and higher IgG antibody responses to whole-*Pg *sonicate antigens than healthy control participants and tended to have higher *Pg *antibody reactivity than patients with other CTDs. In early RA patients prior to DMARD therapy, *Pg *antibody responses correlated significantly with anti-CCP antibody reactivity and, to a lesser degree, with erythrocyte sedimentation rate (ESR) values. Moreover, there was a trend toward higher disease activity and more functional impairment in patients with *Pg *antibodies, and these trends remained during 12 months of DMARD therapy. Our observations are consistent with a role for *Pg *in disease pathogenesis in a subset of RA patients.

## Patients and methods

### Early rheumatoid arthritis cohort study and other subject groups

Newly diagnosed adult patients with early RA were recruited from the Rheumatology Clinic at Massachusetts General Hospital (MGH). The study was approved by the MGH Institutional Review Board, and written consent was obtained from each participant. Patients with disease of less than RA of one year's duration who had not yet received methotrexate (MTX) or biologic DMARD treatment were eligible to participate. Study physicians calculated a classification criteria score for each patient referred to the study, based on clinical assessment, electronic medical record (EMR) review or both. Only those patients with a score ≥6/10 were included, as required by the 2010 rheumatoid arthritis classification criteria promulgated by the American College of Rheumatology/European League Against Rheumatism Collaborative Initiative [17]. For comparison, sera were obtained from patients with long-standing RA seen in the MGH Rheumatology Clinic. Sera were also collected from patients with other CTDs who were evaluated in the MGH Rheumatology Clinic or identified from the MGH Clinical Immunology Laboratory. For healthy control groups, sera were collected from blood bank donors and from healthy hospital personnel. Hospital personnel did not report a history of RA or PD and were similar in age to those in the early RA group. However, clinical examinations for PD were not performed at the time of the study for any of the participants.

### Clinical assessment

Clinical and laboratory assessments of the early RA patients were performed at study entry and at 12-month follow-up visits. Demographic data, including smoking history, were recorded at the time of enrollment. At each study visit, patients completed a Health Assessment Questionnaire (HAQ), and they marked a self-assessment of global health on a 10-cm Visual Analogue Scale. Study physicians performed a clinical assessment, including a 28-joint count, and they marked the physician's global disease score on a 10-cm Visual Analogue Scale. This information was recorded in the patient's EMR using the rheumatology-specific, informatics-based application Rheumatology OnCall (ROC), which was previously developed in our department [18]. The DAS28-ESR value was automatically calculated in the ROC application, which integrates institutional laboratory values for ESR and CRP with physician-entered swollen and tender joint counts and patient global DAS for each date of visit. Clinical Disease Activity Index (CDAI) score was calculated by study physicians using information from the ROC application. Study physicians treated patients according to the standard of care.

### Laboratory determinations

Serum samples were collected at each visit. RF and anti-CCP antibodies were measured at the time of study entry in the MGH Clinical Immunology Laboratory by nephelometry (IMMAGE 800; Beckman Coulter, Brea, CA, USA) and enzyme-linked immunosorbent assay (ELISA) (QUANTA Lite CCP3 IgG ELISA kit; INOVA Diagnostics, San Diego, CA, USA), respectively. ESR and CRP values were determined at each visit by MGH Clinical Laboratories. Serum samples from each visit were frozen at -70°C for subsequent determinations, including *Pg *antibody levels.

*Pg *antibody responses were determined in our laboratory by ELISA using a sonicate preparation of whole, formalin-fixed *Pg *ATCC 33277 (American Type Culture Collection, Manassas, VA, USA) [19]. On the day prior to testing, 96-well polystyrene ELISA plates (Easy Wash; Corning, Tewksbury, MA, USA) were coated with *Pg *antigens (diluted 1:50) and incubated overnight at 4°C. On the next day, each well was incubated with 200

l of blocking buffer (5% nonfat dry milk in phosphate-buffered saline and Tween 20) at room temperature for 1 h. Following washing between each step, wells were incubated at room temperature with 100

l of each of the following: the patient's serum sample (diluted 1:400) and goat anti-human IgG conjugated to horseradish peroxidase (1:2,000), in each instance for 1.5 h, and then tetramethylbenzidine for 5 min, followed by sulfuric acid. The absorbance was measured at 450 nm using an ELISA plate reader (Model 550; Bio-Rad Laboratories, Hercules, CA, USA). To control for interassay variation, each assay included eight serial dilutions of a reference positive control sample as well as eight negative control samples, and all samples from the same patient were included on the same plate. A positive antibody response was defined as >2 SD above the mean absorbance of the 19 age-similar healthy hospital personnel. *Pg *antibody responses were not determined until after completion of the 12-month study period, and therefore treatment decisions were not influenced by these results.

### Statistical analyses

*Pg *geometric mean antibody levels were compared between groups by performing an unpaired *t*-test with the Welch correction. Categorical data were analyzed using Fisher's exact test. Median values for clinical parameters in early RA patients were compared using the Mann-Whitney *U *test. Correlations between *Pg *antibody values and clinical parameters were performed using Pearson's correlation test. All statistical analyses were performed with GraphPad Prism version 5.01 software (GraphPad Software, La Jolla, CA, USA). A *P *value <0.05 was considered statistically significant. All *P *values are two-tailed.

## Results

### Clinical characteristics of patient groups

The 50 patients with early RA--the primary study group--were typical of a cohort of patients with this disease (Table 1). They had a median age of 52 years, were predominantly female (72%) and the majority (84%) had rheumatoid factor (RF) or anti-CCP antibodies, or both. At the time of study entry, they had a median disease duration of 4 months, and their ESR and CRP values, disease activity scores (DAS28-ESR), tender and swollen joint counts, CDAI and HAQ results encompassed the range from mild to severe disease. Only a minority (34%) were current or former smokers. Some patients were receiving nonsteroidal anti-inflammatory drugs or low-dose prednisone, but, by definition, none had yet received MTX or biologic DMARDs.

**Table 1:** Clinical findings at study entry in patients with early rheumatoid arthritis^a^

Parameters	Data (*N *= 50)
Demographics	
Age, years (range)	52 (30 to 80)
Sex, females/males	36/14
Smoking history, *n *(%)	
Current	8 (16)
Former	9 (18)
Never	33 (66)
Disease duration, months	4 (1 to 12)
Autoantibodies	
Rheumatoid factor (RF), no. positive (%)	30 (60)
Anti-CCP	39 (78)
RF or anti-CCP	42 (84)
Inflammatory indices	
Erythrocyte sedimentation rate (ESR), mm/h (range)	23 (3 to 98)
C-reactive protein (CRP), mg/L (range)	7.6 (0.2 to 118)
Disease activity	
DAS28-ESR	4.7 (1.6 to 8.1)
Tender joint count (TJC), *n *(range)	8 (0 to 26)
Swollen joint count (SJC), *n *(range)	3 (0 to 23)
Clinical Disease Activity Index (CDAI)	19.2 (2 to 57.7)
Health Assessment Questionnaire (HAQ)	0.35 (0 to 2.55)
Medications prior to study entry, *n *(%)	
Prednisone	13 (26)
Nonsteroidal anti-inflammatory drug (NSAID)	30 (60)
Methotrexate (MTX)	0
Tumor necrosis factor (TNF) inhibitor	0

^a^Data are expressed as median (interquartile range) values unless otherwise noted. Anti-cyclic citrullinated peptide: anti-CCP; DAS28: Disease Activity Score based on 28-joint count.

For comparison, serum samples were collected from patients with late RA or other CTDs and from healthy control participants (Table 2). The 43 patients with late RA had a median disease duration of 9.5 years (range, 1 to 50 years). Most were seropositive for RF or anti-CCP antibodies (86%). The majority were present or former smokers (70%), and nearly all (96%) were receiving DMARD therapy. Another CTD group included 17 patients with lupus, and a third group consisted of 10 patients with Sjögren syndrome, 4 with scleroderma and 3 with mixed CTDs. Two healthy control groups were included. One consisted of healthy hospital personnel, and the other included a larger group of blood bank donors.

**Table 2:** Age and sex of the early rheumatoid arthritis group and comparison groups^a^

			Patients			
**Demographic categories**	**Age-similar** **hospital** **personnel** **(*N *= 19)**	**Blood bank donors** **(*N *= 53)**	**Systemic** **lupus erythematosus** **(*N *= 17)**	**Other** **CTD** **(*N *= 17)**	**Early** **RA** **(*N *= 50)**	**Late** **RA** **(*N *= 43)**

Age, mean (range)^b^	48 (36 to 80)	N/A	44 (25 to 59)	50 (27 to 88)	53 (30 to 80)	60 (21 to 85)
Sex, females/males^c^	7/12	N/A	17/17	15/17	36/14	31/12

^a^CTD: connective tissue disease; N/A: not available; RA: rheumatoid arthritis. ^b^For comparison of age in early RA group vs. late RA group, *P *= 0.01; for early RA vs. systemic lupus erythematosus, *P *= 0.003. There were no other significant differences in age among the groups. ^c^Regarding sex distribution, healthy hospital personnel, who were primarily men, were significantly different from each of the patient groups, which were composed primarily of women (*P *≤ 0.01). The members of the lupus group, which was exclusively female, were significantly different than both RA patient groups (*P *< 0.05).

By definition, healthy hospital personnel were selected because they were of similar age to those in the early RA group. Patients in the third CTD group also had similar ages as the individuals in these groups. However, compared with the early RA group, the lupus patients were significantly younger (*P *= 0.003) and those with late RA were significantly older (*P *= 0.01). As is typical of these rheumatic diseases, the RA patients and other CTD patients were predominantly female and the lupus patients were exclusively female. The majority of age-similar hospital personnel were men. No demographic or clinical information about blood bank donors was available.

### *Porphyromonas gingivalis *immunoglobulin G antibody responses

**Figure 1:**
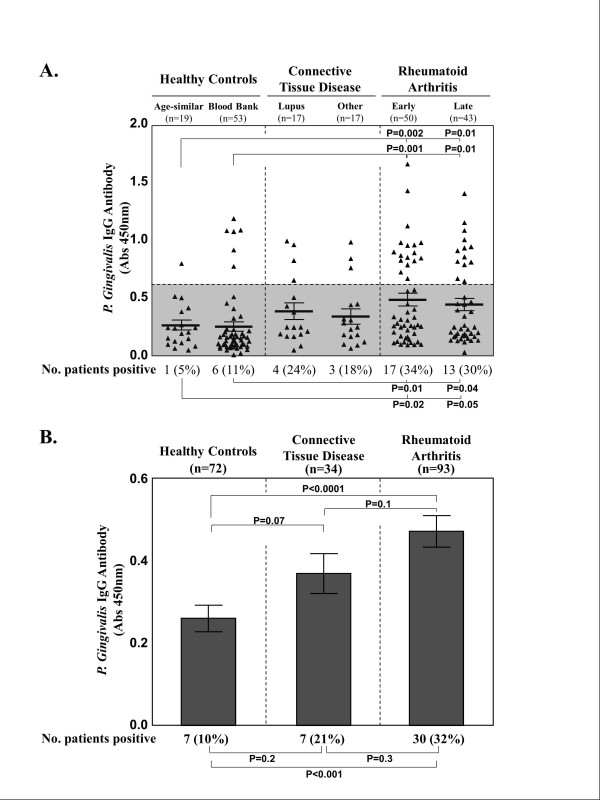
***Porphyromonas gingivalis *immunoglobulin G antibody responses**. *Porphyromonas gingivalis *(*Pg*) antibody responses are shown in age-similar healthy hospital personnel and blood bank donors, in patients with early or late RA and in patients with other connective tissue diseases (CTDs), including 17 patients with lupus and another group consisting of 10 patients with Sjögren syndrome, 4 with scleroderma and 3 with mixed CTD. **(A) **All data points are shown for each individual group. **(B) **The results are summarized, and data from all healthy control participants and patients with CTD or RA are combined. In (B), the histograms give the mean values, and I-bars show the standard error of the mean. The shaded region represents <2 SD above the mean absorbance of healthy hospital personnel. Abs: absorbance; IgG: immunoglobulin G.

At study entry, the frequency of positive *Pg *IgG antibody responses and the magnitude of these responses were highest in RA patients, intermediate in those with other CTDs and lowest in the healthy control groups (Figure 1). Of the 50 patients with early RA, 17 (34%) had positive IgG antibody responses to *Pg*, as did 13 (30%) of the 43 patients with late RA (Figure 1A). Compared with age-similar hospital personnel and blood bank donors, *Pg *positivity in the early RA patients was significantly greater in frequency (*P *= 0.02 and *P *= 0.01, respectively) and *Pg *antibody levels were of significantly greater magnitude (*P *= 0.002 and *P *= 0.001, respectively). Similarly, compared with either group of healthy participants, positive *Pg *IgG responses in late RA patients were also more frequent (*P *= 0.05 and *P *= 0.04, respectively) and the levels were of greater magnitude (in both instances, *P *= 0.01). Patients with lupus or other CTDs also had less frequent *Pg *antibody responses and lower levels than both RA groups, but greater responses than healthy control participants. However, these differences between individual groups were not statistically significant.

When similar groups were combined, RA patients (early and late RA patients) had a significantly greater mean *Pg *antibody response than healthy control participants (blood bank donors and age-similar healthy control individuals) (*P *< 0.0001) (Figure 1B). Moreover, RA patients had higher mean *Pg *antibody responses than patients with CTD (lupus and other CTDs) (*P *= 0.1), and CTD patients had a higher mean *Pg *response than healthy control participants (*P *= 0.07), which were differences of possible significance.

### Clinical correlation of *Porphyromonas gingivalis *antibody responses with anti-CCP antibodies and other standard RA biomarkers

**Figure 2:**
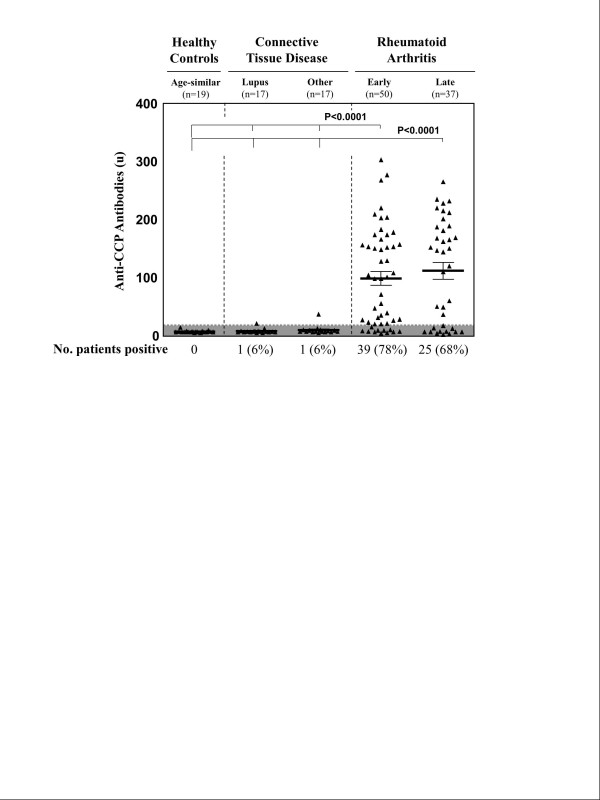
**Anti-cyclic citrullinated peptide antibody levels in rheumatoid arthritis patients and comparison groups**. Anti-cyclic citrullinated peptide (anti-CCP) antibody responses are shown in age-similar healthy hospital personnel, patients with connective tissue diseases and early and late rheumatoid arthritis patient groups. Mean values and standard errors of the mean are shown, and the shaded region represents negative values.

Consistent with typical RA cohorts, 39 (78%) of the 50 patients with early RA had anti-CCP antibody responses, as did 25 (68%) of the 43 patients with late RA (Figure 2). In contrast, only 1 (6%) of the 17 patients with lupus and 1 (6%) of the 17 patients with another CTD had borderline low-level anti-CCP antibody reactivity. Of the two anti-CCP antibody-positive patients with other CTD, the one with lupus was thought to have a lupus-RA overlap syndrome and the other had scleroderma but not arthritis. Neither patient had positive *Pg *antibody responses. None of the 19 age-similar healthy control participants had CCP antibody responses. These differences between RA patients and the other groups were highly significant (*P *< 0.0001).

**Figure 3:**
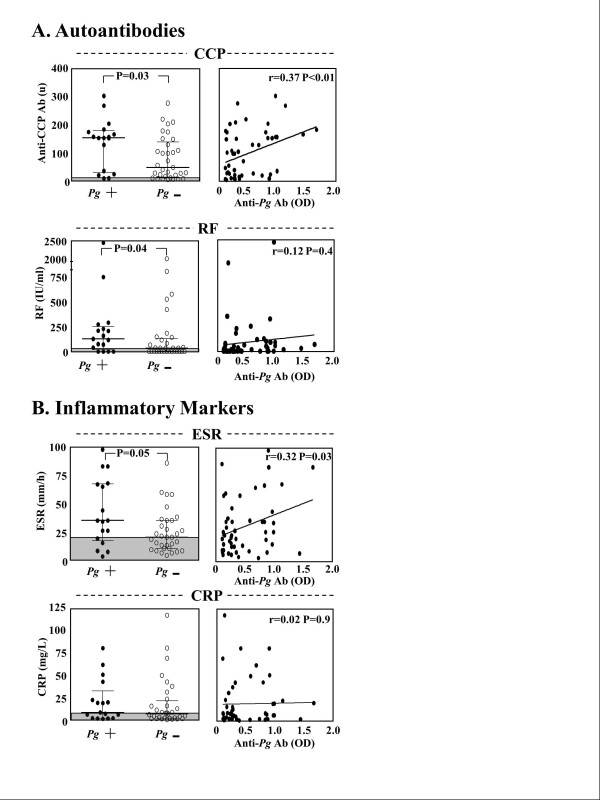
**Correlation of *Porphyromonas gingivalis *antibody levels and standard rheumatoid arthritis biomarkers**. In the patients with early rheumatoid arthritis (RA), the levels of autoantibodies (anti-cyclic citrullinated peptide (anti-CCP) **(A) **and rheumatoid factor (RF)) and inflammatory markers (erythrocyte sedimentation rate (ESR) and C-reactive protein (CRP)) **(B) **were compared at study entry in the 17 patients with positive *Porphyromonas gingivalis *(*Pg*) antibody responses and in the 33 patients with negative *Pg *antibody reactivity, as shown in the left panels. In the right panels, the levels of each RA biomarker are correlated with *Pg *immunoglobulin G antibody levels (Pearson correlation coefficients). Median values and interquartile ranges are shown, and the shaded regions represent normal values. Ab: antibody; OD: optical density.

In the early RA patients, the primary study group, *Pg *antibody responses were correlated with anti-CCP antibody reactivity and other standard RA biomarkers. At the time of study entry, 15 (88%) of the 17 early RA patients with positive *Pg *antibody responses had elevated CCP antibody levels compared with 24 (72%) of the 33 patients with negative *Pg *antibody responses (*P *= 0.3). Moreover, CCP antibody levels were significantly higher in patients with positive *Pg *antibody responses than in those who lacked *Pg *reactivity (*P *= 0.03), and the levels of *Pg *antibodies correlated directly with anti-CCP levels (*r *= 0.37, *P *< 0.01) (Figure 3A). Similarly, 13 (76%) of the 17 early RA patients with positive *Pg *antibody responses had positive results for RF, compared with 18 (55%) of the 33 patients who did not have *Pg *antibodies (*P *= 0.2). Although RF values were higher in those with *Pg *antibody reactivity (*P *= 0.04), there was no correlation between the magnitude of RF and *Pg *antibody responses.

In addition, 13 (76%) of the 17 patients with positive *Pg *antibody responses had elevated ESR levels compared with 18 (55%) of the 33 patients who lacked *Pg *antibodies (*P *= 0.2). Furthermore, ESR values were significantly higher in patients with positive *Pg *antibody responses (*P *< 0.05), and ESR levels correlated with *Pg *antibody levels (*r *= 0.32, *P *= 0.03) (Figure 3B). Patients with positive *Pg *antibody responses also had higher CRP values, but the magnitude of these values did not correlate significantly with *Pg *antibody responses. Overall, although most patients had positive test results for RF and anti-CCP autoantibodies and elevated inflammatory markers, the values were higher in the group with *Pg *antibodies.

### Measurements of disease activity and function prior to and after disease-modifying antirheumatic drug therapy

**Figure 4:**
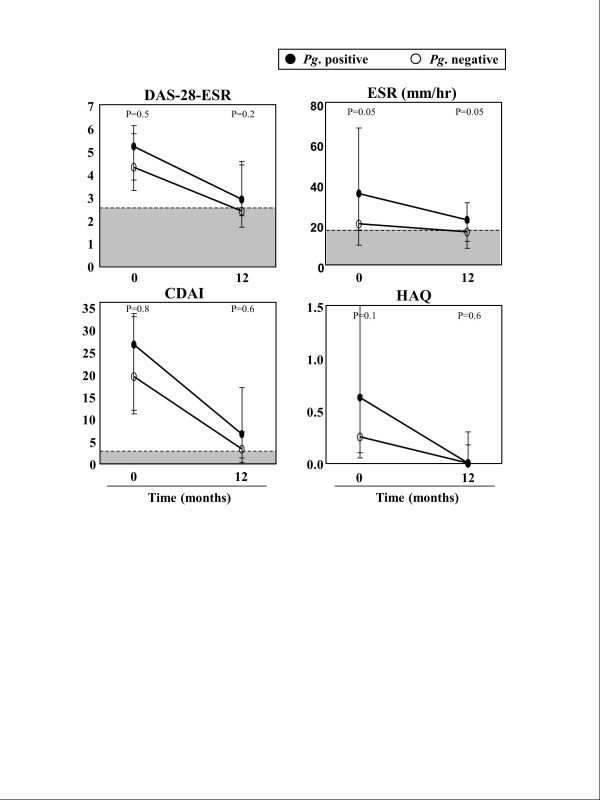
**Disease activity prior to and after 12 months of disease-modifying antirheumatic drug treatment**. Disease activity scores and functional measures ( Disease Activity Score based on 28-joint count erythrocyte sedimentation rate (DAS28-ESR), ESR, Clinical Disease Activity Index (CDAI), and Health Assessment Questionnaire (HAQ)) are shown according to *Porphyromonas gingivalis *(*Pg*) antibody status at the time of study entry, prior to disease-modifying antirheumatic drug therapy and after 12 months of treatment. Data were available at the time of study entry for all 17 *Pg*-positive and all 33 *Pg*-negative patients. At the 12-month follow-up visits, data for DAS28-ESR, ESR and CDAI were available from 16 *Pg*-positive and 22 *Pg*-negative patients. For the HAQ, data were available from 12 *Pg*-positive and 22 *Pg*-negative patients. Median values and interquartile ranges are shown, and the shaded regions represent normal or negative values. Although disease activity measures declined in both groups during the 12-month period, patients with *Pg *antibodies tended to have higher values, primarily for ESR and DAS28-ESR.

At the time of study entry, prior to DMARD therapy, there was a trend toward higher disease activity scores (DAS28-ESR) in early RA patients with positive *Pg *antibody responses. Additionally, ESR values were significantly greater in those with positive *Pg *antibodies (Figure 4). The CDAI levels were also slightly higher in the *Pg *antibody-positive group. HAQ scores, a functional measure, also tended to be higher (indicating a greater degree of dysfunction) in patients with positive *Pg *antibody responses than in those with negative *Pg *responses (median values = 0.63 vs. 0.25; *P *= 0.1). Moreover, the degree of dysfunction correlated directly with the magnitude of *Pg *antibody reactivity (*r *= 0.3, *P *= 0.05; data not shown). Surprisingly, a history of past or present smoking tended to be more common among patients who lacked *Pg *antibody responses than in those who had *Pg *reactivity (42% vs. 18%; *P *= 0.1). Furthermore, compared with patients who lacked *Pg *antibodies, those with positive *Pg *antibody responses were more often treated with prednisone prior to study entry (41% vs. 18%; *P *= 0.1), presumably because of more severe disease.

After their first visit, all 50 patients with early RA were treated with DMARDs, usually MTX, and in some cases TNF inhibitors. During the following 12-month period, *Pg *antibody levels remained the same or declined slightly (data not shown), and no patient with an initial negative *Pg *antibody response developed a positive response during the study period. Conversely, the ESR and disease activity measures (DAS28-ESR and CDAI) generally improved, and functional scores (HAQ) usually returned to normal. However, compared with patients who lacked *Pg *antibody responses, those with positive *Pg *responses still tended to have higher DAS28-ESR scores and significantly higher ESR values at the 12-month follow-up visit (Figure 4). Moreover, at 12 months, only 6 (38%) of the 16 patients who had positive *Pg *antibody responses achieved DAS28 remission compared with 14 (64%) of the 22 patients who lacked *Pg *antibodies (*P *= 0.2), though this trend was not statistically significant. Thus, in patients with elevated *Pg *antibodies, the trend toward greater disease activity, as measured by DAS28 scores, remained after 12 months of DMARD therapy.

## Discussion

In this study, 34% of patients with early RA, prior to DMARD therapy, had positive IgG antibody responses to *Pg*. These antibody responses were of significantly greater frequency and magnitude than those of age-similar healthy hospital personnel or blood bank donors. Most of the RA patients were women, and the majority of hospital personnel were men. Although men may carry a greater risk of PD than women [20], a highly significant difference was still shown between the RA patients and healthy participants. In addition, *Pg *antibody levels in early RA patients were similar to or even higher than those in late RA patients, even though the latter group had more risk factors for PD, including older age and more frequent smoking history [21]. Furthermore, the similar or higher *Pg *antibody levels in DMARD-naïve early RA patients compared with DMARD-treated late RA patients suggest that immunosuppression from DMARD therapy did not alter or enhance *Pg *antibody reactivity. Although 26% of the early RA patients at the time of enrollment were receiving steroids, which may potentially lower antibody levels, *Pg *antibody levels tended to be higher in patients receiving steroids, suggesting that steroid treatment did not decrease *Pg *antibody levels.

We also included a comparison group consisting of patients with other CTDs typically associated with systemic inflammation and autoantibody production, including lupus, mixed CTD, Sjögren syndrome or scleroderma. About 20% of these patients had positive antibody responses to *Pg*, which was a lesser percentage than RA patients (34%) but greater than age-similar healthy control participants (5%). We hypothesize that *Pg *reactivity in patients with other CTD may have been caused by PD, nonspecific immune stimulation or both. However, because almost all of the patients with other CTDs lacked anti-CCP antibodies, cross-reactivity with citrullinated proteins does not appear to account for increased *Pg *reactivity in the non-RA patient groups. Thus, we conclude that a subset of RA patients seen early in the illness prior to DMARD therapy have positive *Pg *IgG antibody responses, which were more frequent and of higher magnitude than those in individuals in the other groups we tested.

Determining which *Pg *antigens to use for serologic tests is problematic. Using whole-*Pg *lysates, a *Pg*-specific lipopolysaccharide or recombinant citrullinated *Pg *PAD, other investigators have previously demonstrated increased frequencies of *Pg *antibody responses in patients with chronic RA compared with healthy control participants [5,13-16,22], as we did using whole-sonicate antigens. In the only previous study of new-onset RA patients, antibody responses to *Pg *specific chaperone protein HtpG were not significantly different from those in healthy control participants [4]. However, antibody responses to this protein may protect against PD and may even be higher in healthy subjects [23]. In our study, we chose to use whole-sonicate antigens because little is known about the frequencies of reactivity with specific *Pg *antigens, even in patients with PD. Therefore, the use of whole-sonicate antigens, which encompass many *Pg *proteins, would increase the possibility of detecting responses. Although such preparations would presumably also increase the potential for false-positive results because of cross-reacting antibodies to other organisms or cross-reactivity between bacterial and host citrullinated proteins [24], we attempted to address this issue by testing serum samples from multiple comparison groups. Future serologic studies using multiple types of assays, such as ELISA and Western blot analysis, as well as different antigen preparations, including noncitrullinated *Pg *proteins, will be important to define further the sensitivity and specificity of *Pg *antibody responses in RA patients.

In our study, a limitation in our ability to interpret *Pg *antibody responses is the lack of dental examinations in case and control participants. In our study, the cutoff value for a positive response was 2 SD above the mean value in the age-similar healthy control participants who reported no history of PD. Yet, it is possible that some of these individuals had PD, which might obscure a difference between RA patients and control participants. However, a highly significant difference was still shown between these groups. Others have previously noted an increased frequency of PD in RA patients [1-5]. *Pg *antibody responses correlate well with the presence of PD [25], and higher *Pg *antibody titers and DAS28 scores have been noted in RA patients with more severe PD [5]. However, *Pg *antibodies may also be associated with a host protective response or carriage of *Pg *as a commensal opportunistic pathogen [23,26]. Therefore, in future studies, it will be important to perform *Pg *antibody studies along with dental examinations, as we have now initiated in our cohort.

In addition to RA, increased frequencies and severity of PD have been reported in several other rheumatic diseases, including ankylosing spondylitis [27], scleroderma [28] and psoriatic arthritis [29]. Although patients with Sjögren syndrome have xerostomia, periodontitis does not seem to be more frequent in these patients [30]. In addition to *Pg *reactivity, antibody responses to several other PD pathogens have been found in RA patients, including *Prevotella intermedia*, *Prevotella melaninogenica*, *Actinobacillus actinomycetemcomitans *and *Bacteroides forsythus *[13,16]. Thus, among rheumatic diseases, increased frequencies of PD may not be unique to RA and increased frequencies of positive antibody responses to PD pathogens may include organisms other than *Pg*.

What does seem to be unique in RA is the association between *Pg *and anti-CCP antibody responses. Consistent with previous reports [14,15], our early RA patients with *Pg *antibodies more often had anti-CCP antibody reactivity. Their anti-CCP responses were significantly higher, and the levels of anti-*Pg *antibodies correlated directly with anti-CCP levels. In contrast, anti-CCP antibody responses were rarely found in patients with other CTDs, and, if present, the levels were very low. Moreover, these responses were not present in healthy control participants. Mikuls *et al*. recently noted that *Pg *antibodies may be present prior to the development of synovitis [31], and, in our study, no patient who initially had negative results for *Pg *antibodies developed these antibodies during 12 months of DMARD therapy. Taken together, these observations suggest that immunity to *Pg *is one factor that may set the stage for autoimmunity and inflammatory synovitis in a subset of RA patients.

Citrullination of proteins may occur within the joint as well as at other sites of inflammation, such as the lung and periodontium [32,33]. In our study, *Pg*-negative RA patients were more likely to be smokers, suggesting that the lung, rather than the gingiva, may have been the site of protein citrullination in these patients. Further studies which examine the lung and periodontium in RA patients are needed to evaluate this hypothesis. Although *Pg *may be involved in RA pathogenesis through citrullination, there may be other important mechanisms by which *Pg *contributes to disease activity in RA. For example, the organism may be more likely to skew CD4+ T-cell reactivity to a Th17 phenotype [34], a response which has been implicated in autoimmunity [35].

Despite specific mechanisms that may account for the association of *Pg *immunity with RA, we found, as others have [5,14,16], that RF, general markers of inflammation (ESR), scores of disease activity (DAS28 and CDAI) and functional impairment (HAQ) were also greater in patients with *Pg *antibody responses. Moreover, the trends remained for the DAS28-ESR and ESR values during 12 months of DMARD therapy. Although the CDAI scores were also slightly higher in the *Pg *antibody-positive group, the differences were not as great as those in the DAS28-ESR, suggesting that ESR is an important contributor to differences in disease activity scores between the groups. Thus, *Pg *antibodies may simply be a marker for PD, a chronic inflammatory condition that may itself be associated with elevation of inflammatory markers. However, the trend toward more severe disease activity in *Pg *antibody-positive patients using indices (HAQ and CDAI) that do not incorporate inflammatory markers suggests that *Pg *itself may contribute to RA disease activity.

There are possible confounding issues in the association of *Pg *antibody reactivity with greater disease activity in RA. For example, certain health behaviors, such as lack of routine dental care, might extend to noncompliance with RA treatment. Factors not studied here, such as the shared epitope (SE), may contribute to the severity of PD [36] as well as RA, and the SE has been associated with periodontal bone destruction in RA patients [37]. However, other studies in RA patients have not found a correlation between the SE and PD or *Pg *antibodies [5,14]. Because of the heterogeneous environmental and host factors involved in RA, patient cohorts may vary, accounting for inconsistent results in clinical correlations among studies [4,5,14-16]. However, consistent with our results, there is general agreement that *Pg *immunity is associated with greater inflammation in RA patients.

In a preliminary study of patients with established RA, Ortiz *et al*. reported that disease activity, as measured by DAS28 score and swollen joint counts, improved with nonsurgical treatment of PD [38]. *Pg *antibodies as a marker for PD may prove to be useful in the identification of patients who would benefit from such treatments. It will be particularly important to conduct studies of therapies for PD and *Pg *in patients with early RA because PD treatment may make the most difference in early stages of the disease.

## Conclusions

A subset of early DMARD-naïve RA patients had positive *Pg *antibody responses which were more frequent and of higher magnitude than those in patients with other CTDs or healthy control participants. Compared with patients who lacked *Pg *antibodies, those with positive *Pg *responses more often had anti-CCP antibody reactivity, their anti-CCP levels were significantly higher and the levels of anti-*Pg *antibodies correlated directly with anti-CCP levels. Furthermore, there was a trend toward higher disease activity and more functional impairment in patients with *Pg *antibody responses, and these trends remained during 12 months of DMARD therapy. These findings are consistent with a role for *Pg *in disease pathogenesis in a subset of RA patients.

## Abbreviations

ACR: American College of Rheumatology; Anti-CCP: anti-cyclic citrullinated peptide; CDAI: Clinical Disease Activity Index; CRP: C-reactive protein; CTD: connective tissue disease; DAS28: Disease Activity Score based on 28-joint count; DMARD: disease-modifying antirheumatic drug; EULAR: European League Against Rheumatism; ELISA: enzyme-linked immunosorbant assay; ESR: erythrocyte sedimentation rate; HAQ: Health Assessment Questionnaire; MGH: Massachusetts General Hospital; MTX: methotrexate; NSAID: nonsteroidal anti-inflammatory drug; PAD: peptidylarginine deiminase; PD: periodontal disease; *Pg*: *Porphyromonas gingivalis*; RA: rheumatoid arthritis; RF: rheumatoid factor; ROC: Rheumatology OnCall; SD: standard deviation; SE: shared epitope; Th: T helper.

## Competing interests

The authors declare that they have no competing interests.

## Authors' contributions

SLA performed the clinical data collection, conducted the laboratory research experiments, performed data interpretation and analysis, contributed to the study design and was responsible for the writing of the manuscript. DSC, MCF, SU and GLC participated in the selection, follow-up and medical care of enrolled patients and reviewed the manuscript. GAM, TK and KS assisted with the design and conduct of the laboratory experiments and reviewed the manuscript. AS, as senior author, designed and coordinated the study and contributed to interpretation of the data and the intellectual input in drafting the manuscript. All authors read and approved the final manuscript.
